# Transcriptional regulatory network for the establishment of CD8^+^ T cell exhaustion

**DOI:** 10.1038/s12276-021-00568-0

**Published:** 2021-02-24

**Authors:** Wooseok Seo, Chandsultana Jerin, Hiroyoshi Nishikawa

**Affiliations:** 1 Department of Immunology, Nagoya University Graduate School of Medicine, Nagoya, 466-8550 Japan; 2 Division of Cancer Immunology, Research Institute/Exploratory Oncology Research and Clinical Trial Center (EPOC), National Cancer Center, Tokyo/Chiba, Japan

**Keywords:** Gene regulation in immune cells, CD8-positive T cells

## Abstract

Chronic infection with persistent antigenic stimulation results in the generation of exhausted CD8^+^ T cells, which are considered defective effector CD8^+^ T cells, and thus compromises effective immune responses. However, recent studies have illustrated that exhausted CD8^+^ T cells may be purposely generated and maintained to provide mild immune responses against chronic infection or cancer, which can be safer over a long period of time than strong immune responses. Indeed, a specific population of exhausted CD8^+^ T cells that behaves similarly to self-renewing stem cells and provides a continuous supply of exhausted CD8^+^ T cells has been identified, indicating that this population can be considered progenitors of exhausted CD8^+^ T cells. Furthermore, several ground-breaking studies in the last few years have shed new light on the transcriptional regulatory network governing the generation and propagation of exhausted CD8^+^ T cells, which involves T cell receptor (TCR) signaling that leads to NFAT-TCF1 (nuclear factor of activated T cells-T cell factor 1) activity followed by activation of the TOX/NR4A axis. Elucidation of the intracellular signaling pathways will help to define the definitive developmental stages leading to exhausted CD8^+^ T cells, which can be exploited to advance our never-ending battle against cancer. This review will summarize the recent discoveries that have deepened our understanding of the exhaustion program of cytotoxic CD8^+^ T cells.

## Immunity: exhausted T cells provide subtle long-term defenses

A type of T cell previously thought to be defective could actually provide long-term mild immune responses for combating cancer. The unique environment created during chronic infections gives rise to functionally inert T cells known as ‘exhausted’ cells, which were once thought to impede immune responses. Wooseok Seo and co-workers at Nagoya University and the National Cancer Center in Tokyo, Japan, have reviewed recent studies suggesting that exhausted T cells may be generated by the body to provide mild immune responses. Such responses are safer over the long term than strong inflammatory defense mechanisms, where T cells may be overstimulated, leading to uncontrolled tissue damage and induced T cell death. The signaling pathways that generate and propagate exhausted cells are being revealed, shedding light on the developmental stages that lead to this subtle but effective response to chronic conditions.

## Introduction

T cell progenitors leave the bone marrow and migrate to the thymus, where they undergo a series of maturation steps and selection processes to give rise to mature helper CD4^+^ or cytotoxic CD8^+^ T cells^1^. Mature naïve (antigen-unexperienced) T cells enter the periphery, where they continuously migrate among the secondary lymphoid organs through the blood and lymphatic circulation. Within the secondary lymphoid organs, such as the lymph nodes, naïve T cells receive survival signals to maintain their homeostasis, such as weak T cell receptor (TCR) signaling from self-peptides loaded on major histocompatibility complexes (MHCs) or cytokine signaling from IL-7/IL-15^2^. Additionally, the secondary lymphoid organs provide places where naïve T cells can physically interact with professional antigen-presenting cells (APCs) and scan the surfaces of APCs for the presence of cognate antigens^3^. Upon encountering and recognizing antigens through their TCRs with appropriate strength, naïve T cells are stimulated to become effector T cells and exert their function as part of adaptive immunity^4^. In the case of cytotoxic CD8^+^ T cells, stimulation of naïve CD8^+^ T cells (KLRG1^-^IL-7Rα^+^) results in the upregulation of killer cell lectin-like receptor G1 (KLRG1) expression and downregulation of IL-7Rα (CD127) expression^5^. These early effector T cells (KLRG1^+^IL-7Rα^lo^) exponentially proliferate and differentiate further into short-lived effector cells (SLECs) that exhibit a terminally differentiated state (KLRG1^hi^IL-7Rα^lo^) with the full features of cytotoxicity. Therefore, KLRG1 expression has been considered a marker of the differentiation steps of effector CD8^+^ T cells^6^. Once a pathogen is successfully cleared, effector CD8^+^ T cells quickly die by AICD during the contraction phase of immune responses, which prevents stimulated CD8^+^ T cells from damaging surrounding healthy tissues.

A few effector CD8^+^ T cells evade AICD and survive by downregulating KLRG1 expression and re-expressing IL-7α (KLRG1^-^IL-7Rα^+^). This so-called exKLRG1 population forms memory precursor effector cells (MPECs) and generates long-lived memory CD8^+^ T cells^7^. Interestingly, some naïve CD8^+^ T cells never express KLRG1 and become KLRG1^-^IL-7Rα^lo^ MPECs, indicating that there are at least two different pathways leading to MPECs. The extracellular signals and intracellular signaling pathways leading to the bifurcation process of terminally differentiated effector and memory CD8^+^ T cells are not fully understood but are an active area of research^6,8^. Regardless of their complicated differentiation pathways, circulating memory CD8^+^ T cells in the human body can be classified into two major populations: effector memory T cells (T_EM_; KLRG1^lo^IL-7Rα^+^CD44^+^CD62L^-^), which are abundant in recently infected tissues; and central memory CD8^+^ T cells (T_CM_; KLRG1^lo^IL-7Rα^+^CD44^+^CD62L^+^), which consistently recirculate through the lymphoid organs. Contrary to acute infection, chronic infection creates a unique environment that leads CD8^+^ T cells into an alternative differentiation pathway and generates functionally inert CD8^+^ T cells, which have been termed ‘exhausted cells’. These cells have been shown to contribute to immune dysfunction in numerous chronic infections and cancers, making them popular targets for clinical intervention. We will review recent findings about the mechanisms governing CD8^+^ T cell exhaustion from the perspective of the transcriptional regulatory network.

## Hyporesponsiveness of T cells

**Fig. 1:**
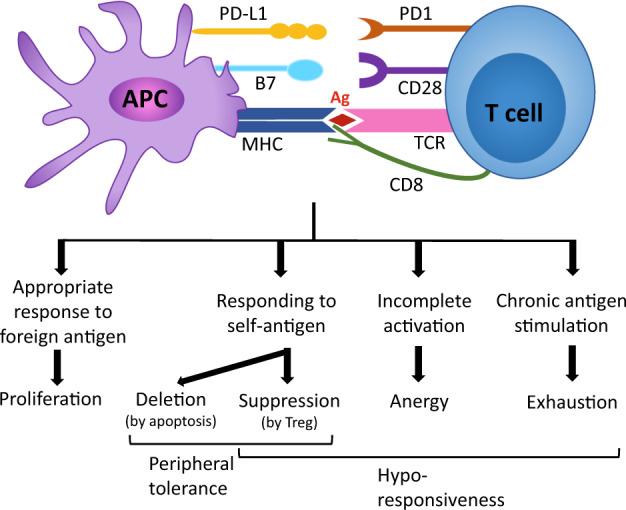
Hyporesponsiveness of CD8^+^ T cells. Naïve cytotoxic CD8^+^ T cells scan the cell surface of antigen-presenting cells (APCs), such as dendritic cells (DCs), which present processed peptides on MHC molecules. The inputs from T cell receptors (TCRs) function as signal 1, those from costimulatory molecules (such as CD28, OX40, and ICOS) act as signal 2, and cytokines (such as IL-12) provide signal 3; these signals are combined to generate appropriate intracellular signaling events that dictate the outcome of T cell fate. Recognition of cognate foreign antigens with appropriate signals 2 and 3 activates CD8^+^ T cells to differentiate into effector T cells. CD8^+^ T cells responding to self-antigens are subjected to peripheral tolerance, in which they are either deleted by apoptosis or inactivated (or suppressed) by regulatory T cells (Tregs). Insufficient signals 2 and 3 or strong negative signals provided by coinhibitory molecules (such as PD1 and CTLA4) result in incomplete activation of T cells, which are then called anergic T cells. Persistent cognate antigen stimulation during chronic infection or cancer induces another hyporesponsive state of T cells called exhaustion, which is characterized by high expression of multiple coinhibitory molecules.

Naïve T cells are stimulated by cognate antigens and differentiate into specific subsets of effector T cells, but not all effector T cells are equally functional. Some effector T cells show attenuated activity and less proliferation as well as a decreased response to secondary stimulation^9^. Such a hyporesponsive (or dysfunctional) state of T cells is not just a failure of the immune system; in some cases, at least, it appears to be a normal process to protect the body from uncontrolled excessive immune responses by effector T cells^10^. We can classify this hyporesponsive state of T cells largely into tolerance, anergy and exhaustion (or dysfunction)^11,12^ based on the causes of its occurrence (Fig. 1). Since these hyporesponsive states have been extensively discussed and reviewed elsewhere, we will only briefly describe them here.

The first class of T cell hyporesponsiveness is T cell tolerance, which is an active mechanism that suppresses T cells responding to self-antigens, and it acts both on T cell progenitors in the thymus (central tolerance)^13^ and mature T cells (peripheral tolerance)^14^. Central tolerance is the main mechanism of negative selection against developing T cell progenitors in the thymus, and it eliminates T cell progenitors that are reactive to self-antigens. Since not all self-antigens are available during the negative selection process, T cells that could potentially be reactive to self-antigens exist in the periphery. Therefore, our immune system also has peripheral tolerance, which keeps these potentially self-reactive T cells in the periphery in check by either deleting (apoptosis) them or suppressing them, which prevents autoimmune diseases. Anergy is the second class of T cell hyporesponsiveness and is induced by overwhelming coinhibitory signals. An anergic state can be induced in vitro by stimulating T cells without costimulatory signals or in vivo with suboptimal antigenic stimulation^15^. Since anergy is induced in some T cells due to self-reactive TCRs, anergy can also be considered a part of peripheral tolerance (Fig. 1). The last class of T cell hyporesponsiveness, which is the subject of this review, is called exhaustion, which phenotypically resembles anergy; however, recent studies have shown that anergic and exhausted T cells are quite distinct populations^11^. T cell exhaustion is induced after repeated and prolonged antigenic stimulation during chronic infection. Contrary to acute infection, chronic infection provides a unique environment within which effector T cells differentiate into exhausted T cells rather than memory T cells. Therefore, it has been implied that continuous availability of antigens is required for the development and maintenance of exhausted T cells, but recent studies suggest that there may be a specific progenitor population of exhausted T cells that can actively maintain exhausted T cell pools even without the persistent presence of antigens^16^. Further studies will be required to decipher the requirement for antigens in the maintenance of the exhausted T cell pool.

## T cell exhaustion

The concept of immune exhaustion has been known for a long time, but it was systemically characterized as a specific state of T cell responsiveness just in the last 20 years. Exhausted T cells are perceived as T cells in unresponsive and nonfunctional states during chronic virus infection because the term ‘exhaustion’ was first used to describe the reduced capacity of effector CD8^+^ T cells to secrete inflammatory cytokines, such as IFNγ and TNF, as well as cytotoxic effector molecules, such as granzyme and perforin, all of which are hallmarks of cytotoxic effector T cells^17^. Another distinctive feature of exhausted CD8^+^ T cells is the extensive expression of multiple coinhibitory molecules. Normal effector CD8^+^ T cells express several inhibitory receptors, such as PD1 and CTLA4, during the usual course of activation, which balances immune responses induced with costimulatory receptors, such as CD28 and ICOS. However, exhausted CD8^+^ T cells show elevated and prolonged expression of many coinhibitory receptors, including LAG3, 2B4 (CD244), CD160, TIM3, and TIGIT, in addition to PD1 and CTLA4, which can easily overwhelm positive costimulatory signals and tip the balance toward exhaustion^18^. Additionally, CD8^+^ T cell exhaustion is tightly linked with metabolism, and exhausted CD8^+^ T cells exhibit a unique metabolic state. Naïve CD8^+^ T cells acquire energy mainly from the mitochondria-driven tricarboxylic acid (TCA) cycle through oxidative phosphorylation (OXPHOS)^19,20^, but effector CD8^+^ T cells heavily rely on aerobic glycolysis to meet their massive energy requirements^21,22^. On the other hand, inhibitory receptor ligation on exhausted T cells induces signaling events that suppress AKT activation and mTOR activity, resulting in a switch from glycolysis to fatty acid oxidation (FAO)^23,24^. This metabolic reprogramming of exhausted CD8^+^ T cells from glycolysis to lipolysis is believed to be a main survival mechanism of exhausted CD8^+^ T cells, especially under the restricted availability of nutrients common in chronic infection or cancer^25^.

**Fig. 2:**
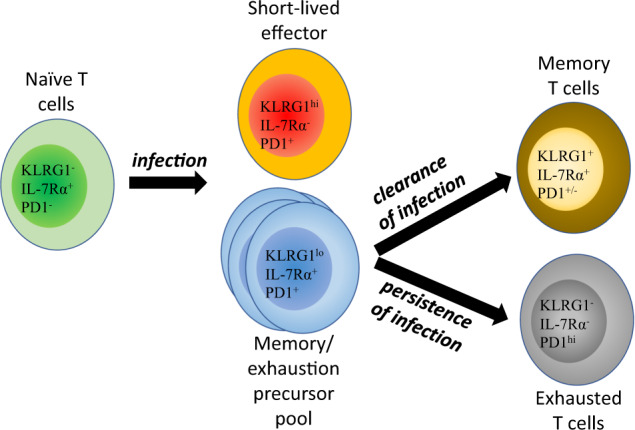
Differentiation of exhausted T cells. T cell activation generates both short-lived effector cells with terminally differentiated features and memory precursors. If the infection is cleared without delay (acute infection), the short-lived effector cells die, and the memory precursors give rise to long-lived memory T cells. However, if the infection persists without resolution (chronic infection), the memory precursors generate PD1^+^CXCR5^+^TCF1^+^ progenitors of exhausted T cells that self-renew to maintain the pool of mature exhausted T cells (PD1^+^TCF1^-^). It appears that these progenitors of exhausted T cells phenotypically resemble memory progenitors during acute infection, but they functionally differ in the generation of daughter cells.

A large body of studies regarding CD8^+^ T cell exhaustion has been performed in the context of viral infection since acute or chronic infection can be deliberately induced. For example, lymphocytic choriomeningitis virus (LCMV) causes either an acute or chronic infection depending on the specific virus strain, with the Armstrong strain used for acute infection and Clone 13 used for chronic infection. More recently, it was found that CD8^+^ T cells within the tumor microenvironment (TME) resemble exhausted CD8^+^ T cells in chronic infection since abundant ligands for coinhibitory receptors and an environment in which aerobic glycolysis is difficult are generated^26^. Additionally, CD8^+^ T cells responding to cancer cells typically react to tumor antigens with low avidity since tumor antigens are derived from self-peptides that are weakly immunogenic at best. Moreover, many types of cancer cells can downregulate MHCI molecule expression, which impairs the antigen presentation required for appropriate CD8^+^ T cell activation. Therefore, chronic infection and cancer can stably persist over a long time due to the fine balance between pathogenesis and host immune responses^27^. This has raised an intriguing question about how effector CD8^+^ T cells in the exhausted state can be maintained for months or even years, and it was hypothesized that exhausted CD8^+^ T cells may be able to self-renew from a progenitor-like population (Fig. 2). It was even suggested that exhausted CD8^+^ T cells may purposely develop from normal effector CD8^+^ T cells because attenuated immune responses can be potentially less harmful than excessive effector functions during the long fight against infections or cancers^28^.

The exact ontogeny of exhausted CD8^+^ T cells remains incompletely elucidated currently, and after all, there may be more than one developmental pathway, which is supported by the high heterogeneity of exhausted CD8^+^ T cells^29^. Many studies regarding the generation of exhausted CD8^+^ T cells have thus employed coinhibitory receptors that can be used as surface markers of exhausted T cells. Since coinhibitory receptors are functional molecules, blocking one or multiple coinhibitory receptors has now become a promising approach to treat various cancers. However, the clinical efficacy of this approach is still quite limited since the detailed downstream signaling pathways that lead to the exhaustion program of CD8^+^ T cells are unclear. We believe that understanding the transcriptional network of the exhaustion program is critical; indeed, it was shown that many features of exhausted CD8^+^ T cells, such as impaired production of effector molecules, are associated with altered usage of several transcription factors including T-bet (T-box expressed in T cells), EOMES (eomesodermin), and BLIMP1 (B lymphocyte-induced maturation protein-1). This implies that transcriptional and epigenetic programs need to be clearly defined to better understand the molecular ontogeny of exhausted CD8^+^ T cells^30^. Below, we will describe the transcription factors that have been reported to be involved in T cell exhaustion, but it should be noted that many transcription factors shown to be involved in CD8^+^ T cell exhaustion are not exclusive to the exhaustion program, as they are also used in other cellular functions.

## T-bet and EOMES

Activated CD8^+^ T cells express the transcription factors T-bet and EOMES, both of which are essential for effector functions^31,32^. T-bet and EOMES play redundant roles, but their expression levels are somewhat different in effector and memory CD8^+^ T cells, indicating that functional specificities must exist between T-bet and EOMES^6^. In short, early effector CD8^+^ T cells (T-bet^+^EOMES^+^) gradually increase T-bet expression to become terminally differentiated effectors (T-bet^++^EOMES^+^), whereas memory CD8^+^ T cells have higher expression of EOMES (T-bet^+^EOMES^++^), indicating that the reciprocal expression of T-bet and EOMES influences the fate of early effector CD8^+^ T cells, which can become either terminal effector CD8^+^ T cells or memory CD8^+^ T cells^33^. However, if an infection is not cleared and becomes chronic, effector CD8^+^ T cells (T-bet^++^EOMES^+^) reduce T-bet expression but further increase EOMES expression (T-bet^+^EOMES^+++^), which executes the exhaustion program instead of the generation of memory CD8^+^ T cells.

It is not entirely clear how the differential expression of T-bet and EOMES can determine the fate of effector CD8^+^ T cells, but it appears that PD1 expression is correlated with the fate decision process since terminal effector T cells have low PD1 expression (T-bet^++^Eomes^+^PD1^+^), whereas exhausted CD8^+^ T cells have high PD1 expression (T-bet^+^EOMES^+++^PD1^++^)^34^. This correlation was confirmed genetically in a paper by Paley et al., which showed that deletion of EOMES resulted in reductions in the levels of exhaustion markers, including PD1, and perturbation of the exhaustion process, thus proving that upregulation of EOMES expression is closely related to the terminal differentiation of exhausted CD8^+^ T cells^29^. On the other hand, CD8^+^ T cells with conditional knockout of T-bet show increased expression of coinhibitory receptors, including PD1, and forced expression of T-bet produces the opposite result^35^. Therefore, genetic evidence supports the conclusion that the differential levels of T-bet and EOMES control the fate decisions leading to effector versus exhausted T cell generation. Interestingly, it appears that not all early effector CD8^+^ T cells (T-bet^+^EOMES^+^) in chronic infections become exhausted CD8^+^ T cells (T-bet^+^EOMES^+++^); some remain to proliferate continuously, thus maintaining the supply of exhausted CD8^+^ T cells over a long period of time. These progenitors of exhausted CD8^+^ T cells can be reinvigorated by PD1/PD-L1 blockade, which is suggestive of the involvement of reversible epigenetic changes operating within the progenitor population of exhausted CD8^+^ T cells. Overall, T-bet and EOMES together with PD1 execute the actual exhaustion program, but the generation and maintenance of the progenitor pool relies on other transcription factors, such as TCF1 (T cell factor 1), as we describe below.

## TCF1

Two recent companion publications reported that during chronic infection, PD1^+^CD8^+^ T cells consisted of two distinct populations that could be divided according to CXCR5 expression^36,37^. CXCR5 is known as the receptor for CXCL13, which mediates B cell homing into the follicles in the secondary lymphoid organs. Therefore, all mature B cells express CXCR5, and activated helper CD4^+^ T cells also express it to access the B cell zone (named as follicular helper T cells). The existence of a small population of CXCR5^+^CD8^+^ T cells was also known, but their function was not clear until these two recent papers. He et al. showed that in the context of chronic viral infection, approximately one-third of virus-specific effector CD8^+^ T cells expressed CXCR5, with these cells exhibiting higher costimulatory molecule expression but fewer coinhibitory molecules (including PD1) than CXCR5^-^CD8^+^ T cells, suggesting that CXCR5 expression is associated with less exhausted states. Im et al. further illustrated that the transcriptome of the CXCR5^+^CD8^+^ population was enriched in signaling molecules related to Wnt pathways, which is associated with the stem cell-like self-renewal capacity, indicating that the CXCR5^+^ population exhibits precursor-like properties. Taken together, these studies propose that PD1^+^CXCR5^+^CD8^+^ T cells represent the precursor population of exhausted CD8^+^ T cells during chronic infection.

Interestingly, Im et al. further discovered that PD1^+^CXCR5^+^CD8^+^ T cells expressed the T cell-specific transcription factor TCF1 (encoded by *Tcf7*) during the early phase of chronic infection, whereas PD1^++^CXCR5^-^CD8^+^ T cells did not express much TCF1, indicating that TCF1 during the early phase of chronic infection might be associated with the progenitor status of exhausted CD8^+^ T cells. In fact, TCF1 deletion resulted in the absence of a PD1^+^CXCR5^+^ population and substantially reduced generation of exhausted T cells. The importance of TCF1 for the generation of PD1^+^CXCR5^+^CD8^+^ progenitor cells in early chronic infection was further confirmed by another study performed with TCF1 reporter mice, which showed that TCF1-expressing CD8^+^ T cells maintained immune responses during chronic infection and exhibited characteristics of both memory CD8^+^ T cells and exhausted CD8^+^ T cells^38^. Previously, TCF1 was shown to be highly expressed in T cell progenitors in the thymus and naïve CD8^+^ T cells in the periphery but expressed at lower levels in effector CD8^+^ T cells^39–41^. Therefore, these new studies propose a new role for TCF1 in the generation of progenitors of exhausted CD8^+^ T cells^42,43^. However, it should be noted that TCF1 also plays a similar role in the generation and maintenance of progenitor populations of memory CD8^+^ T cells during acute infection.

There is one interesting recent study showing that the expression of PD1 is important to the role of TCF1^44^. Chen et al. showed that KLRG1^+^PD1^-^ cells lacked TCF1 expression and instead expressed T-bet, which is indicative of an effector-like phenotype. On the other hand, KLRG1^-^PD1^+^ cells expressed TCF1 and EOMES and exhibited reduced proliferation, which is indicative of exhaustion. In fact, conditional deletion of TCF1 favored the differentiation of KLRG1^+^ effector-like CD8^+^ T cells, whereas enforced expression of TCF1 suppressed KLRG1^+^ cells. These results not only reconfirm a previous report showing the critical requirement for TCF1 in the generation of exhausted CD8^+^ T cells but also indicate that PD1 expression is required to reinforce the function of TCF1 during the induction of exhaustion programs. This new research sheds new light on the molecular mechanism by which PD1 exerts its function as an inhibitory receptor. Further studies will be required to understand how much PD1 contributes to executing the exhaustion program in vivo.

## NFAT

During the early phase of chronic infection, the progenitors of exhausted CD8^+^ T cells are generated and maintained by the TCF1 transcription factor, and the exhaustion program is gradually achieved by the cooperation of T-bet, EOMES, and PD1, as we described above. However, one of the important questions during the onset of the exhaustion program is how acute versus chronic infection is decided, in other words, how relevant information is transmitted from TCRs to intracellular signaling pathways. It is well known that the transcription factor NFAT (nuclear factor of activated T cells) interacts with its partner Fos-Jun (AP1) transcription factors to form a complex that is indispensable for CD8^+^ T cell activation and a wide variety of effector functions. Engagement of TCRs and costimulatory CD28 receptors generates signaling cascades resulting in the activation of NFAT and AP1, thus translating extracellular signals into intracellular pathways through TCR/CD28. Thus, it is plausible to expect that NFAT/AP1 also influences T cell hyporesponsiveness through TCR/CD28 signaling.

How the NFAT/AP1 complex affects the decision process for effector function versus exhaustion is not completely clear currently, but some evidence suggests crucial roles for NFAT. For example, it was shown that the expression of NFAT (specifically that of NFAT2) is specifically upregulated in exhausted CD8^+^ T cells, whereas that of AP1 is downregulated^45^. This is quite different from the pattern in effector CD8^+^ T cells, in which both NFAT and AP1 levels are elevated. This is a particularly interesting discovery since it has been shown that the ratio of NFAT to AP1 is important during the decision process for effector function versus anergy^46^. Therefore, it is possible that the balance between NFAT and AP1 may be an important factor during the onset of the exhaustion process. In fact, a recent study suggested that NFAT can promote T cell exhaustion by binding DNA regions independently of AP1. Specifically, NFAT directly binds to the regulatory regions of coinhibitory receptor genes, including *Pdcd1* (PD1) and *Havcr2* (TIM3), thus enforcing the exhaustion program^47,48^.

**Fig. 3:**
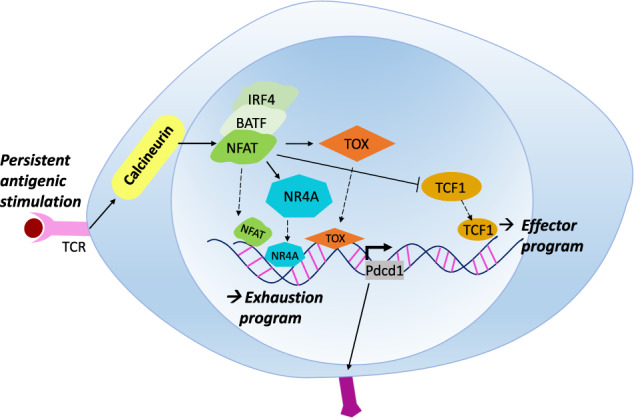
Transcriptional regulatory network of exhausted CD8^+^ T cells. T cell receptor (TCR) activation triggers phosphorylation of the kinase Lck, which in turn activates calcineurin pathways. Calcineurin dephosphorylates cytoplasmic nuclear factor of activated T cells (NFAT), which then translocates into the nucleus and regulates genes involved in either T cell effector function or T cell exhaustion. Therefore, the transduction of extracellular signals by NFAT is one of the most important steps in T cell exhaustion. On the one hand, NFAT represses TCF1 in exhausted CD8^+^ T cells since TCF1 can induce the formation of terminal effector and memory CD8^+^ T cells by upregulating EOMES expression. On the other hand, NFAT induces the transcription factors TOX and NR4A, which execute the exhaustion program of CD8^+^ T cells. It should be noted that TCF1 is necessary in the progenitors of exhausted T cells, but it should be repressed in terminally differentiated exhausted T cells.

Adding yet another layer of complexity to our understanding of the NFAT-mediated exhaustion program, it has been shown that the transcription factors IRF4 (interferon regulatory factor 4) and BATF (basic leucine zipper ATF-like transcription factor) together with NFAT participate in the exhaustion of CD8^+^ T cells induced in response to TCR-mediated signaling^49^. This study showed that NFAT is required for the activation of the transcription factors BATF and IRF4 and that this triplet of transcription factors (NFAT/IRF4/BATF) plays important roles in mediating the CD8^+^ T cell exhaustion program. Especially, the authors provided evidence that NFAT/IRF4/BATF tightly represses TCF1 in exhausted CD8^+^ T cells since TCF1 should be absent from terminally differentiated exhausted CD8^+^ T cells even though it is required during early progenitor stages. In summary, these studies reveal that TCR/CD28 signals are transduced to activate NFAT/IRF4/BATF, which suppresses TCF1 in terminally differentiated exhausted CD8^+^ T cells (Fig. 3).

## TOX and NR4A

As we described above, TCR/CD28 signals are transmitted to NFAT, which pushes the TCF1^+^ progenitor population toward a terminally differentiated exhausted CD8^+^ T cell phenotype. However, previously it was not clear how NFAT and TCF1 can execute the exhaustion program because they lack expression specificity. A series of ground-breaking papers published in 2019 finally identified a network of transcription factors specifically induced during chronic infection that execute the exhaustion program. Alfei et al. showed that in the context of chronic infection with LCMV Clone 13, CD8^+^ T cells specifically induced the expression of TOX, the thymocyte selection-associated high-mobility group box^50^. The transcription factor TOX is expressed by peripheral CD8^+^ T and NK cells as well as T cell progenitors in the thymus, but the molecular mechanisms by which TOX exerts its function have been difficult to decipher since high-mobility group box proteins, including TOX, bind DNA in a sequence-independent way^51^. TOX germline-knockout mice completely lack helper CD4^+^ T cells, indicating that TOX is required for the proper development of helper-lineage T cells^52^. Further studies have shown that TOX induces the expression of ThPOK, a lineage-specifying factor of helper CD4^+^ T cells, and helps MHCII-restricted cells establish a CD4^+^ T cell lineage gene program^53^. Therefore, it was quite surprising to find that deletion of TOX in the hematopoietic lineage by using *Mx1-cre* resulted in perturbed generation of exhausted CD8^+^ T cells under chronic infection. TOX deletion specifically resulted in the downregulation of PD1 expression and increases in the expression of cytotoxic effector molecules, suggesting that the exhaustion program was severely inhibited. Also, the absence of TOX also resulted in a reduction in the TCF1^+^TIM3^-^ progenitor population of exhausted CD8^+^ T cells, indicating that TOX plays a role in the early phase of the exhaustion program. Together with the direct correlation between the expression level of TOX and immunopathology, these data collectively suggest that TOX drives genetic programs during the initiation and maintenance of T cell exhaustion. This discovery further implied that T cell exhaustion is not the unfortunate outcome of chronic infection and is instead a well-controlled functional adaption of effector CD8^+^ T cells. Since TCR signaling is transduced into NFAT signaling that can induce TOX, the transcriptional regulatory network of exhausted CD8^+^ T cells can be summarized as TCR-NFAT-TCF1-TOX^54,55^. These studies have further shown that the TOX-mediated execution of the exhaustion program is not only restricted to chronic infection but also operates in tumors^56^. In the absence of TOX, exhausted CD8^+^ T cells in the TME cannot be maintained, which results in enhanced cancer eradication, indicating that TOX prevents CD8^+^ T cells from becoming exhausted.

Interestingly, analysis of transcriptomes obtained from exhausted CD8^+^ T cells found that NR4A (nuclear receptor group 4 family A) showed an expression pattern similar to that of TOX, suggesting that the transcription factors TOX and NR4A might play related roles in the induction of the CD8^+^ T cell exhaustion program downstream of NFAT^57^. NR4A has three family members, NR4A1 (Nru77), NR4A2 (Nurr1), and NR4A3 (NOR-1), and they have been shown to play numerous roles in immune cells, especially in the development and activation of regulatory T (Treg) and invariant natural killer T (iNKT) cells^58,59^. During the development of T cells, NR4A participates in the negative selection process of T cell progenitors with strong affinity for self-antigens, thus implementing central tolerance in the thymus. However, the roles of NR4A proteins in other lymphoid-lineage cells, especially CD8^+^ T cells, have not been resolved. Liu et al. discovered that NR4A expression was highly upregulated in in vitro-generated exhausted CD8^+^ T cells in concert with TOX^60^. The authors found strong interplay between TOX and NR4A, indicating that their cooperation might be important during the exhaustion program. Additionally, TOX and NR4A appeared to be directly upregulated by NFAT, thus establishing the TCR-NFAT-TCF1-TOX/NR4A axis^61^. Indeed, ablation of NR4A1 further produced increased effector function and decreased exhaustion features. Specifically, exhausted CD8^+^ T cells from *Nr4a1*^-/-^ mice showed decreased levels of inhibitory receptors such as PD1 and TIM3, leading to enhanced anticancer immunity in various mouse cancer models. Surprisingly, the authors found that the NR4A1 binding sites in the genome significantly overlapped with those of AP1, indicating that NR4A1 might antagonize AP1-mediated transcriptional programs, which are associated with T cell activation. Therefore, it appears that NR4A helps to commit cells to the exhaustion program by strengthening NFAT signaling, as exhaustion requires more NFAT than AP1 (see section NFAT above).

## Concluding remarks

Recent studies strongly propose that exhaustion of CD8^+^ T cells provides a regulatory mechanism to prevent CD8^+^ T cells from being overstimulated, which can lead to uncontrolled tissue damage and activation-induced cell death. Therefore, the suppression of the effector functions of CD8^+^ T cells by exhaustion is necessary in responses to chronic infection and cancer. However, most studies on exhausted CD8^+^ T cells have been conducted at the population level, and there is only limited information on how an individual CD8^+^ T cell is fated between effector/memory function and exhaustion^62,63^. Therefore, more studies using single-cell technologies will allow deciphering of the relationships of memory cell progenitors during acute infection and exhausted cell progenitors during chronic infection.

The newly revealed molecular pathway of the CD8^+^ T cell exhaustion program including the TCR-NFAT-TCF1-TOX/NR4A axis exhibits enormous potential for the development of new anticancer therapies, but we are still confronted with several critical questions that await further studies. One intriguing question is the extent to which TOX and NR4A functionally overlap, and whether these transcription factors physically interact to cooperate during the execution of the exhaustion program. Another fundamental question regarding CD8^+^ T cell exhaustion in chronic settings is about how the progenitors of exhausted CD8^+^ T cells are maintained. It seems that the exhausted state of CD8^+^ T cells is not fully terminal^64^, and now we know that TCF1^+^ progenitors of exhausted CD8^+^ T cells can be rejuvenated to effector CD8^+^ T cells after PD1 or PDL1 blockade. Can terminally exhausted CD8^+^ T cells be maintained after depriving them of persistent antigenic stimulation? In other words, do terminally exhausted CD8^+^ T cells also need a continuous supply of chronic stimulation? The answer to this question will help us to better understand the relationship between antigen stimulation and the fate reversibility of exhausted CD8^+^ T cells.

## References

[1] Taniuchi I (2018). CD4 helper and CD8 cytotoxic T cell differentiation. Annu. Rev. Immunol..

[2] Sprent J, Surh CD (2011). Normal T cell homeostasis: the conversion of naive cells into memory-phenotype cells. Nat. Immunol..

[3] Neefjes J, Jongsma ML, Paul P, Bakke O (2011). Towards a systems understanding of MHC class I and MHC class II antigen presentation. Nat. Rev. Immunol..

[4] Smith-Garvin JE, Koretzky GA, Jordan MS (2009). T cell activation. Annu. Rev. Immunol..

[5] Joshi NS (2007). Inflammation directs memory precursor and short-lived effector CD8(+) T cell fates via the graded expression of T-bet transcription factor. Immunity.

[6] Kaech SM, Cui W (2012). Transcriptional control of effector and memory CD8+ T cell differentiation. Nat. Rev. Immunol..

[7] Herndler-Brandstetter D (2018). KLRG1(+) effector CD8(+) T cells lose KLRG1, differentiate into all memory T cell lineages, and convey enhanced protective immunity. Immunity.

[8] Sarkar S (2008). Functional and genomic profiling of effector CD8 T cell subsets with distinct memory fates. J. Exp. Med..

[9] Wherry EJ (2011). T cell exhaustion. Nat. Immunol..

[10] Speiser DE (2014). T cell differentiation in chronic infection and cancer: functional adaptation or exhaustion?. Nat. Rev. Immunol..

[11] Schietinger A, Greenberg PD (2014). Tolerance and exhaustion: defining mechanisms of T cell dysfunction. Trends Immunol..

[12] Thommen DS, Schumacher TN (2018). T cell dysfunction in cancer. Cancer Cell.

[13] Kyewski B, Klein L (2006). A central role for central tolerance. Annu Rev. Immunol..

[14] Walker LS, Abbas AK (2002). The enemy within: keeping self-reactive T cells at bay in the periphery. Nat. Rev. Immunol..

[15] Schwartz RH (2003). T cell anergy. Annu Rev. Immunol..

[16] Utzschneider DT (2013). T cells maintain an exhausted phenotype after antigen withdrawal and population reexpansion. Nat. Immunol..

[17] Moskophidis D, Lechner F, Pircher H, Zinkernagel RM (1993). Virus persistence in acutely infected immunocompetent mice by exhaustion of antiviral cytotoxic effector T cells. Nature.

[18] Crawford A, Wherry EJ (2009). The diversity of costimulatory and inhibitory receptor pathways and the regulation of antiviral T cell responses. Curr. Opin. Immunol..

[19] Mendoza A (2017). Lymphatic endothelial S1P promotes mitochondrial function and survival in naive T cells. Nature.

[20] Yang K, Neale G, Green DR, He W, Chi H (2011). The tumor suppressor Tsc1 enforces quiescence of naive T cells to promote immune homeostasis and function. Nat. Immunol..

[21] Wang R (2011). The transcription factor Myc controls metabolic reprogramming upon T lymphocyte activation. Immunity.

[22] Blagih J (2015). The energy sensor AMPK regulates T cell metabolic adaptation and effector responses in vivo. Immunity.

[23] MacIver NJ, Michalek RD, Rathmell JC (2013). Metabolic regulation of T lymphocytes. Annu Rev. Immunol..

[24] Zhang Y (2017). Enhancing CD8(+) T cell fatty acid catabolism within a metabolically challenging tumor microenvironment increases the efficacy of melanoma immunotherapy. Cancer Cell.

[25] Patsoukis N (2015). PD-1 alters T-cell metabolic reprogramming by inhibiting glycolysis and promoting lipolysis and fatty acid oxidation. Nat. Commun..

[26] Baitsch L, Fuertes-Marraco SA, Legat A, Meyer C, Speiser DE (2012). The three main stumbling blocks for anticancer T cells. Trends Immunol..

[27] McLane LM, Abdel-Hakeem MS, Wherry EJ (2019). CD8 T cell exhaustion during chronic viral infection and cancer. Annu Rev. Immunol..

[28] Pauken KE, Wherry EJ (2015). Overcoming T cell exhaustion in infection and cancer. Trends Immunol..

[29] Paley MA (2012). Progenitor and terminal subsets of CD8+ T cells cooperate to contain chronic viral infection. Science.

[30] Wherry EJ, Kurachi M (2015). Molecular and cellular insights into T cell exhaustion. Nat. Rev. Immunol..

[31] Sullivan BM, Juedes A, Szabo SJ, von Herrath M, Glimcher LH (2003). Antigen-driven effector CD8 T cell function regulated by T-bet. Proc. Natl Acad. Sci. USA.

[32] Pearce EL (2003). Control of effector CD8+ T cell function by the transcription factor eomesodermin. Science.

[33] Intlekofer AM (2005). Effector and memory CD8+ T cell fate coupled by T-bet and eomesodermin. Nat. Immunol..

[34] Blackburn SD (2009). Coregulation of CD8+ T cell exhaustion by multiple inhibitory receptors during chronic viral infection. Nat. Immunol..

[35] Kao C (2011). Transcription factor T-bet represses expression of the inhibitory receptor PD-1 and sustains virus-specific CD8+ T cell responses during chronic infection. Nat. Immunol..

[36] Im SJ (2016). Defining CD8+ T cells that provide the proliferative burst after PD-1 therapy. Nature.

[37] He R (2016). Follicular CXCR5- expressing CD8(+) T cells curtail chronic viral infection. Nature.

[38] Utzschneider DT (2016). T cell factor 1-expressing memory-like CD8(+) T cells sustain the immune response to chronic viral infections. Immunity.

[39] Weber BN (2011). A critical role for TCF-1 in T-lineage specification and differentiation. Nature.

[40] Yu Q (2009). T cell factor 1 initiates the T helper type 2 fate by inducing the transcription factor GATA-3 and repressing interferon-gamma. Nat. Immunol..

[41] Zhou X (2010). Differentiation and persistence of memory CD8(+) T cells depend on T cell factor 1. Immunity.

[42] Wu T (2016). The TCF1-Bcl6 axis counteracts type I interferon to repress exhaustion and maintain T cell stemness.. Sci. Immunol..

[43] Leong YA (2016). CXCR5(+) follicular cytotoxic T cells control viral infection in B cell follicles. Nat. Immunol..

[44] Chen Z (2019). TCF-1-centered transcriptional network drives an effector versus exhausted CD8 T cell-fate decision. Immunity.

[45] Wherry EJ (2007). Molecular signature of CD8+ T cell exhaustion during chronic viral infection. Immunity.

[46] Macian F (2002). Transcriptional mechanisms underlying lymphocyte tolerance. Cell.

[47] Martinez GJ (2015). The transcription factor NFAT promotes exhaustion of activated CD8(+) T cells. Immunity.

[48] Agnellini P (2007). Impaired NFAT nuclear translocation results in split exhaustion of virus-specific CD8+ T cell functions during chronic viral infection. Proc. Natl Acad. Sci. USA.

[49] Man K (2017). Transcription factor IRF4 promotes CD8(+) T cell exhaustion and limits the development of memory-like T cells during chronic infection. Immunity.

[50] Alfei F (2019). TOX reinforces the phenotype and longevity of exhausted T cells in chronic viral infection. Nature.

[51] Aliahmad P, Seksenyan A, Kaye J (2012). The many roles of TOX in the immune system. Curr. Opin. Immunol..

[52] Aliahmad P, Kaye J (2008). Development of all CD4 T lineages requires nuclear factor TOX. J. Exp. Med.

[53] Aliahmad P, Kadavallore A, de la Torre B, Kappes D, Kaye J (2011). TOX is required for development of the CD4 T cell lineage gene program. J. Immunol..

[54] Khan O (2019). TOX transcriptionally and epigenetically programs CD8(+) T cell exhaustion. Nature.

[55] Yao C (2019). Single-cell RNA-seq reveals TOX as a key regulator of CD8(+) T cell persistence in chronic infection. Nat. Immunol..

[56] Scott AC (2019). TOX is a critical regulator of tumour-specific T cell differentiation. Nature.

[57] Seo H (2019). TOX and TOX2 transcription factors cooperate with NR4A transcription factors to impose CD8(+) T cell exhaustion. Proc. Natl Acad. Sci. USA.

[58] Sekiya T (2013). Nr4a receptors are essential for thymic regulatory T cell development and immune homeostasis. Nat. Immunol..

[59] Moran AE (2011). T cell receptor signal strength in Treg and iNKT cell development demonstrated by a novel fluorescent reporter mouse. J. Exp. Med.

[60] Liu X (2019). Genome-wide analysis identifies NR4A1 as a key mediator of T cell dysfunction. Nature.

[61] Chen J (2019). NR4A transcription factors limit CAR T cell function in solid tumours. Nature.

[62] Gerlach C (2013). Heterogeneous differentiation patterns of individual CD8+ T cells. Science.

[63] Buchholz VR (2013). Disparate individual fates compose robust CD8+ T cell immunity. Science.

[64] Barber DL (2006). Restoring function in exhausted CD8 T cells during chronic viral infection. Nature.

